# Analysis of the Molecular Mechanisms Governing the Stage-Specific Expression of a Prototypical Housekeeping Gene during Intraerythrocytic Development of *P. falciparum*

**DOI:** 10.1016/j.jmb.2011.02.043

**Published:** 2011-04-29

**Authors:** Eleanor H. Wong, Sandra Hasenkamp, Paul Horrocks

**Affiliations:** 1 Institute for Science and Technology in Medicine, Keele University, Staffordshire ST5 5BG, UK; 2 School of Medicine, Keele University, Staffordshire ST5 5BG, UK

**Keywords:** IDC, intraerythrocytic development cycle, hpi, hours postinfection, RACE, rapid amplification of cDNA ends, EMSA, electrophoretic mobility shift assay, TBP, TATA binding protein, HDAC, histone deacetylase, PCD, programmed cell death, SAHA, suberoylanilide hydroxamic acid, SBHA, suberohydroxamic acid, malaria, posttranscriptional, gene regulation, epigenetic, EMSA

## Abstract

Gene expression during the intraerythrocytic development cycle of the human malarial parasite *Plasmodium falciparum* is subject to tight temporal control, resulting in a cascade of gene expression to meet the physiological demands of growth, replication, and reinvasion. The roles of the different molecular mechanisms that drive this temporal program of gene expression are poorly understood. Here we report the use of the *bxb1* integrase system to reconstitute all aspects of the absolute and temporal control of the prototypical housekeeping gene encoding the proliferating cell nuclear antigen (*Pfpcna*) around an integrated luciferase reporter cassette. A quantitative analysis of the effect of the serial deletion of 5′ and 3′ genetic elements and sublethal doses of histone deacetylase inhibitors demonstrates that while the absolute control of gene expression could be perturbed, no effect on the temporal control of gene expression was observed. These data provide support for a novel model for the temporal control of potentially hundreds of genes during the intraerythrocytic development of this important human pathogen.

## Introduction

*Plasmodium falciparum* is the etiological agent of the most pernicious form of human malaria, a disease that exerts a massive toll on global health, with some 1 million deaths resulting from 247 million cases reported annually.^1^ Following the publication of this organism's genome in 2002,^2^ major inroads have been achieved in our understanding of this parasite's complex biology in both its human host and the anopheline mosquito vector, as well as in describing its pathology at the molecular level.^3^ That said, challenges in the maintenance of an effective drug pipeline and vaccine development underpin the importance of translating this knowledge into an effective control of this pathogen.^4,5^

The life cycle of *P. falciparum* is characterized by its constant adaptation to diverse environments and a strict program of morphological development—with evidence from high-throughput microarray and proteomic analyses indicating that these phenomena are underpinned by a global coordinated pattern of developmentally linked gene expression.^6–8^ This is perhaps best evidenced by the demonstration of a tightly coordinated cascade of coordinated gene expression during the intraerythrocytic development cycle (IDC)^6^—a stage of parasite development that is readily accessible through *in vitro* culture but is also key given that this is the stage of the life cycle that is primarily responsible for the etiology of disease. These data initially suggested a predominant role for cis–trans interactions in directing the temporal control of gene expression, and since this was presumably mediated at the level of transcription initiation, this led to the so-called “transcript-to-go” hypothesis.^6,9^ Subsequent analyses, however, correlating steady-state mRNA levels with peptide profiles have failed to provide proof for this hypothesis.^9–11^ More recently, evidence for developmentally linked mRNA stability, translational repression, and noncoding RNA all suggest that while cis–trans-directed promoter activity may play a major role in the temporal control of gene expression, other molecular mechanisms may equally impact on this process.^12–18^

That said, understanding the contributions that cis–trans interactions in gene flanking sequences make in directing the temporal control of gene expression is of fundamental importance. Early functional data, using transfected reporter constructs, have proven to be key in demonstrating the canonical nature of the *P. falciparum* promoter.^19–24^ Like other eukaryotes, *P. falciparum* promoters are bipartite in nature, where RNA-polymerase-II-mediated transcription from a transcription start site located in a minimal promoter region is directed by cis–trans interactions in flanking sequences.^16,18^ While these studies have proven invaluable in demonstrating a role for these cis–trans interactions in directing the absolute level of transcription, they have proven less so in understanding their role in the temporal control of transcription. This, in general, can be attributed to two aspects of the experimental approaches adopted. The first aspect is the reliance of the reporter constructs on transient transfection; in the absence of drug pressure, plasmids are rapidly lost from the parasite.^25,26^ Moreover, the absence of nucleosome assembly over these plasmids results in what appears to be a constitutive expression of the reporter gene.^26^ Stable episomal transfection overcomes this limitation, as these drug-selected plasmids assemble nucleosomes during S-phase.^27,28^ Using this approach, however, is problematic from a quantitative viewpoint, as these plasmids often concatamerize and show evidence of unequal segregation during mitotic division.^25^ This experimental approach has, however, shown in a small number of cases that appropriate temporal control can be reconstituted for a reporter gene.^29–32^ The mixed success for this approach may be attributed to a second aspect of the approaches adopted: the mix and match of 5′ and 3′ flanking sequences from different genes. Given the potential for both 5′ and 3′ sequences working together to contribute to the temporal regulation of gene expression, this practice may confound any sensible analysis of their relative contributions.

The development of the *bxb1* integrase-mediated transfection system in *P. falciparum* offers an attractive tool to overcome some of these limitations.^33^ In this system, it is claimed that homogeneous populations of genetically modified parasites, each bearing a single chromosomally integrated reporter construct, can be rapidly generated.^33–35^ To this end, we set out here to establish whether such an experimental approach can be adopted to reconstitute the correct temporal control of expression for a luciferase construct from the matched 5′ and 3′ flanking sequences of a *P. falciparum* gene.

The gene selected for this study was *Pfpcna* (Pf13_0328). This gene encodes the proliferating cell nuclear antigen, a key processivity factor for DNA polymerase Δ on the leading strand, accessory roles in DNA replication/repair and maintenance of epigenetic marks during mitosis.^21,36,37^ Three factors directed its choice in this study. First, *Pfpcna* has a known pattern of temporal expression during the IDC; the transcript and protein steady-state levels peak in mature trophozoites, coinciding with the known demand for DNA synthesis.^36^ Second, previous studies have characterized the minimal promoter (including mapping of the transcription start site) and have demonstrated (using a transient transfection system) a role for flanking sequences in directing the absolute level of gene expression.^21^ Third, and perhaps most importantly, *Pfpcna* represents an excellent candidate for a prototypical housekeeping gene under coordinated temporal control during the IDC. *Pfpcna* transcription shares a temporal pattern with over 30 genes known to be involved in DNA replication and metabolism—and more widely with several hundred genes upregulated in expression during the metabolically active trophozoite stage.^6,38^ Over recent years, some excellent work on the control of expression of the different members of the *var* multigene family has been performed—work that reflects the importance of their gene products in the pathology of infected erythrocytes (for reviews, see Llinás *et al.*,^18^ Dzikowski and Deitsch,^39^ Scherf *et al.*,^40^ and Chookajorn *et al.*^41^). However, the temporal control of expression of *var* genes during the IDC is overlaid by a complex layer of control that ensures that only one member of this multigene family is expressed in each parasite and is therefore not typical of the control of the vast majority of the genome.

Here we describe an analysis of the genetic and epigenetic factors that govern the absolute and temporal control of a *P. falciparum* prototypical housekeeping gene in an attempt to describe the molecular mechanisms that govern the control of gene expression in this important human pathogen.

## Results

### Reconstitution of the correct temporal profile of *Pfpcna* expression on an integrated luciferase cassette

The IDC for *P. falciparum* takes 48 h and is characterized by progression through a series of morphologically distinct stages: ring [0–16 h postinfection (hpi)], trophozoite (17–38 hpi), and schizont (39–48 hpi). *Pfpcna* shows a coincident temporal profile of mRNA and protein steady-state levels during the IDC, starting low in rings, peaking in mature trophozoites to meet the demands of DNA synthesis at this stage, and subsiding again in schizonts.^6,21^ Our initial aim was to demonstrate that sufficient *Pfpcna* 5′ and 3′ flanking sequences were used to reconstitute this same temporal pattern of expression for a luciferase reporter cassette integrated into the parasite's genome.

**Fig. 1:**
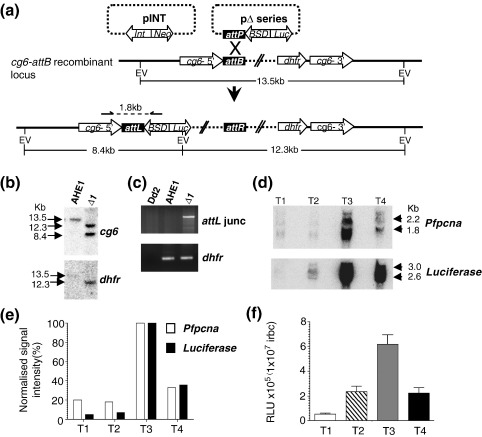
Reconstitution of the correct temporal and absolute control of *Pfpcna* expression in the Δ1 integrant. (a) Site-specific integration of the pΔ series of plasmids into the *att*B–*cg*6 locus of the parasite clone AHE1 facilitated by *bxb1* integrase (*Int*) on pINT. The position of the oligonucleotide probes and the region amplified over the *att*L locus are indicated by inverted arrows and the dotted line. EV, EcoRV restriction site; *BSD*, blasticidin S deaminase selectable marker; *dhfr*, human dihydrofolate reductase selectable marker; *Neo*, neomycin selectable marker. This figure was adapted from Nkrumah *et al.*^33^ (b) Southern blot analysis of 10 μg of genomic DNA restricted with EcoRV from AHE1 and Δ1 and sequentially hybridized with probes to *cg*6 and *dhfr*. Fragment sizes are indicated in kilobases (kb). (c) PCR confirmation of integration in Δ1 by amplification of a 1.88-kb fragment over the *att*L locus generated by a recombination of *att*B and *att*P sites. PCR over 440 bp of *dhfr* acts as control for the maintenance of this site in AHE1. (d) Time-course (T1, ring; T2, early trophozoite; T3, mature trophozoite; T4, schizont) Northern blot analysis of 10 μg of total RNA isolated from Δ1 sequentially hybridized with probes to *Pfpcna* and luciferase genes. (e) Normalization of T1–T4 Northern blot signal intensities for luciferase and *Pfpcna* to signal intensity at T3. (f) Time-course luciferase activity assay data for Δ1 expressed as relative light units (RLU) per 1 × 10^7^ infected red blood cells (irbc). Data represent the mean and standard error of the mean of five independent paired measurements from clones Δ1.1 and Δ1.2.

The plasmid pΔ1 contains a luciferase reporter gene flanked by 1418 bp and 647 bp of 5′ and 3′ *Pfpcna* sequences, respectively, in a pDC*att*P backbone to facilitate integration into the genome of the *P. falciparum* clone AHEI.^33^ In this parasite clone, the *att*B site for the mycobacteriophage *bxb1*'s integration into *Mycobacterium smegmatis* has been inserted into the *cg*6 locus on chromosome 7. Using established transfection and drug selection procedures,^33^ we derived two independent clones, Δ1.1 and Δ1.2. Successful integration of a single copy of pΔ1 into the correct locus was confirmed by PCR over the *att*L site, generated following cross-over recombination between the *att*P site and the *att*B site, as well as Southern blot analysis (Fig. 1a–c).

In order to demonstrate the reconstitution of the correct temporal profile of expression, we carried out serial Northern blot and luciferase assays throughout the IDC. Harvest of RNA and cytoplasmic protein fractions was carried out at the T1 (ring, 6–12 hpi), T2 (early trophozoite, 18–24 hpi), T3 (mature trophozoite, 30–36 hpi), and T4 (schizont, 40–46 hpi) time points. Inspection of parasite morphology on Giemsa-stained thin blood film by light microscopy confirmed the presence of at least 80% of the appropriate stage in each time point. The expected temporal pattern of *Pfpcna* steady-state mRNA levels was observed for the endogenous gene, with transcripts of 1.8 kb and 2.2 kb peaking in level at the T3 (late trophozoite) sample (Fig. 1d).^36^ The luciferase steady-state mRNA levels show a very similar temporal profile, with the peak of expression at T3 (late trophozoite), albeit with transcript sizes of 2.6 kb and 3 kb (Fig. 1d). In order to quantitatively describe the temporal profile, we captured and normalized the signal intensity of the paired transcripts at each stage as a percentage of the peak signal in T3 (Fig. 1e). Comparison of the normalized transcript intensities clearly demonstrates a successful reconstitution of the correct temporal mRNA steady-state levels for luciferase during the IDC. Comparison of the results of the luciferase reporter assays at the equivalent T1–T4 time points similarly demonstrates the reconstitution of a coincident temporal profile of luciferase activity with the expected peak of activity at the T3 late trophozoite stage (Fig. 1f).

**Fig. S1:**
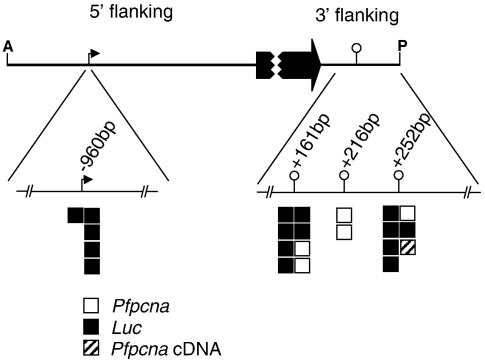
RACE analysis of *Pfpcna* and luciferase transcripts from Δ 1. Five clones corresponding to the 5’ end of the luciferase transcript (black boxes) map to the transcription start site previously mapped for *Pfpcna* 960bp upstream of the open reading frame (ORF). Eleven luciferase and five *Pfpcna* (white boxes) clones corresponding to three polyadenylation sites located 161bp, 216bp and 252bp downstream of the ORF. The hatched box represents a predicted *Pfpcna* polyadenylation site from screening an oligo(dT) primed cDNA library generated from trophozoite RNA (Horrocks and Alano, unpublished data).

The difference in sizes between the transcripts obtained for *Pfpcna* and the transcripts obtained for luciferase (Fig. 1d) was approximately 800 bp, the same difference in size between the open reading frames for the two genes. This would suggest that the transcriptional apparatus recognizes the same transcriptional start and polyadenylation sites in the *Pfpcna* 5′ and 3′ flanking sequences around both the endogenous version and the integrated version of these sequences. *Pfpcna* has a major transcription start site located 960 bp upstream of the open reading frame.^21^ The transcription start site for the luciferase transcript was mapped using 5′ rapid amplification of cDNA ends (RACE), and ClustalN analysis of the five independent clones derived indicated that four of the clones absolutely correlated with the previously mapped *Pfpcna* transcription start site with the last clone's sequence, including an additional eight bases of 5′ sequence (Fig. S1). Due to the lack of information regarding the position of the *Pfpcna* polyadenylation site, 3′ RACE was carried out for both the *Pfpcna* transcript and the luciferase transcript (Fig. S1). A total of 16 clones were generated: 11 were derived from the luciferase transcript, and 5 were derived from the *Pfpcna* transcript. ClustalN analysis revealed two major polyadenylation sites and one minor polyadenylation site. The two major sites (+ 161 bp and + 252 bp from the open reading frame) are apparently utilized by both the *Pfpcna* transcript and the luciferase transcript. The minor site (+ 216 bp) was utilized only by the *Pfpcna* transcript. All three sites are positioned adjacent to a 5′-AATAA-3′ sequence located 10–30 nucleotides upstream of U/GU-rich regions, features predicted to be the consensus sequence for the site of polyadenylation in *Plasmodium* spp.^42,43^ Together, these data indicate that the luciferase transcript from pΔ1 utilizes the *Pfpcna* 5′ and 3′ flanking sequences in determining the limits of the transcript, as well as the absolute and temporal patterns of expression in the same way that these flanking sequences act for the endogenous gene.

### Serial deletions of 5′ and 3′ flanking sequences demonstrate an effect on the absolute, but not temporal, control of *Pfpcna* expression

**Fig. S2:**
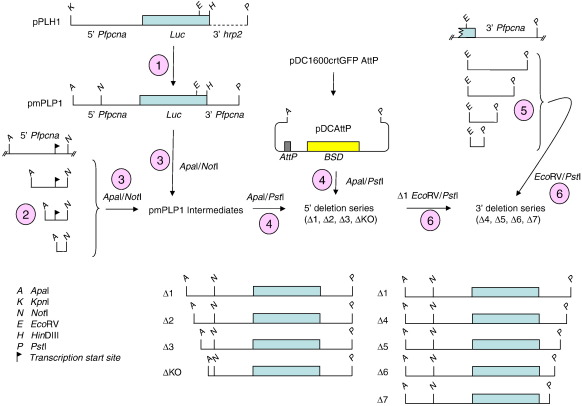
Generation of 5’ and 3’ serial deletion luciferase reporter constructs. A detailed description of steps 1 to 6 is provided in the supplementary materials and methods.

**Fig. S3:**
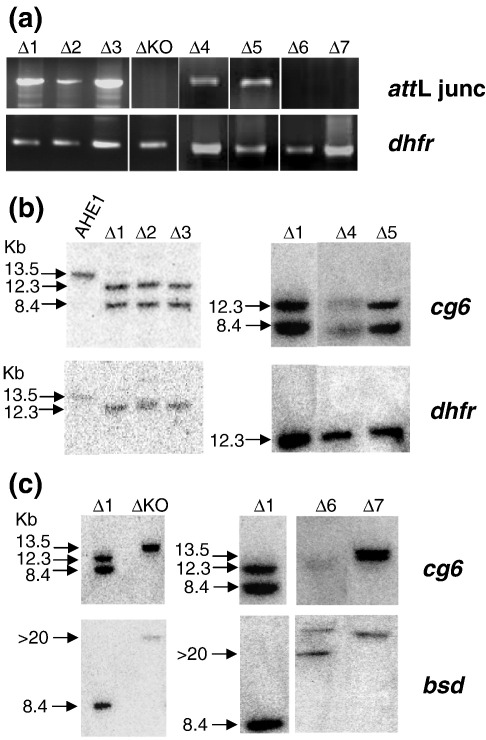
Analysis of 5’ and 3’ deletion series plasmids integration into the *cg6-attB* locus of AHE1 (see also Figure 1a). (A) PCR confirmation of integration in Δ2, Δ3, Δ4 and Δ5 by amplification of 1.88kb fragment over *attL* locus generated by recombination of *attB* and *attP* sites. PCR over 440bp of *dhfr* acts as a control for preparation of genomic DNA suggesting no integration in clones ΔKO, Δ6 and Δ7 (B) Southern blot analysis of 10μg of genomic DNA restricted with EcoRV from parasite clones with an integrated reporter cassette sequentially hybridized with probes to *cg6* and *dhfr*. Fragment sizes are indicated in kilobases (Kb). (C) Southern blot analysis of 10μg of genomic DNA restricted with *EcoRV* from parasite clones where the plasmid construct did not integrate sequentially hybridized with probes to cg6 and bsd. In these clones the *cg6-attB* locus remains intact and the <20kb signal for the pΔ series selectable marker *bsd* indicates the maintenance of episomal concatamerised plasmid.

**Fig. 2:**
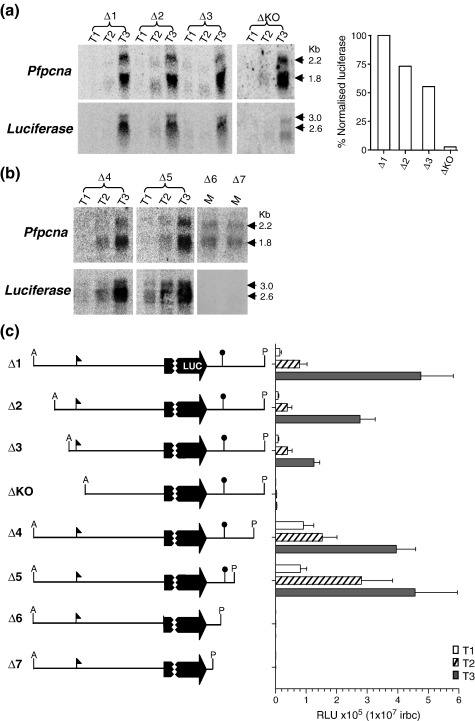
Analysis of absolute and temporal luciferase expressions in 5′ and 3′ *Pfpcna* serial deletion constructs. (a) Time-course (T1, ring; T2, early trophozoite; T3, mature trophozoite/schizont) Northern blot analysis of 10 μg of total RNA isolated from the 5′ deletion series (Δ1-ΔKO) sequentially hybridized with probes to *Pfpcna* and luciferase genes. Transcript sizes are indicated in kilobases (kb). T3 luciferase signal intensities are normalized against matched T3 *Pfpcna* signal intensity and plotted as a proportion of this ratio in Δ1. (b) The first two panels represent time-course (T1–T3) Northern blot analysis of the 3′ deletion series constructs that were successfully integrated (Δ4 and Δ5) and sequentially hybridized with probes to *Pfpcna* and luciferase genes. Mixed (M)-stage total RNA of integrants Δ6 and Δ7 shows the absence of any luciferase transcript signal. (c) The left-hand panel shows the 5′ and 3′ *Pfpcna* flanking sequences around the luciferase reporter cassette for each of the Δ series clones used in this study. Transcription start and polyadenylation sites are indicated with an arrow and a lollipop, respectively. A (ApaI) and P (PstI) restriction sites flank the reporter cassette. The right-hand panel indicates time-course (T1, ring; T2, early trophozoite; T3, mature trophozoite/schizont) luciferase activity assay data for the Δ series expressed as relative light units (RLU) per 1 × 10^7^ infected red blood cells (irbc). Data represent the mean and standard error of the mean of at least five independent paired measurements.

Previous analysis of the 5′ flanking sequences of *Pfpcna* mapped a core promoter region (− 710 bp to − 1180 bp) and a putative enhancer region (− 1250 bp to − 1580 bp).^21^ In order to better delineate sequences in the 5′ and 3′ *Pfpcna* flanking regions that direct the control of both absolute expression and temporal expression, we prepared a series of deletion constructs based on pΔ1 (Fig. 2; Fig. S2) and transfected them into the AHEI clone. As described above, PCR and Southern blot analysis of the resultant transfectants were carried out to demonstrate a correct integration into the *cg*6 locus (Fig. S3). This analysis confirmed the correct integration of pΔ2, pΔ3, pΔ4, and pΔ5, with the remaining constructs (pΔKO, pΔ6, and pΔ7) apparently only maintained episomally as concatamerized plasmid despite four independent attempts at integration. Thus, while these luciferase reporter constructs were present in the parasite, data developed from subsequent luciferase activity assays are only described qualitatively, as they were deemed not equivalent to those transfectants where the plasmid had integrated into chromosome 7.

Northern blot and luciferase reporter assays of samples collected at three time points during the IDC were carried out: T1, ring (6–12 hpi); T2, early trophozoite (18–24 hpi); T3, mature trophozoite/schizont (30–40 hpi). For all the 5′ transfectant clones and for the two 3′ integrated clones, the correct absolute and temporal patterns of *Pfpcna* steady-state mRNA accumulation were observed, with the expected peak in level observed in the mature trophozoite/schizont (T3) stages (Fig. 2a and b). The steady-state mRNA levels for luciferase in Δ2, Δ3, ΔKO, Δ4, and Δ5 clearly mimicked this temporal pattern of gene expression, although there was a notable decrease in the absolute level of luciferase transcript as increasing increments of the 5′ flanking sequence were deleted (Fig. 2a). Normalizing the T3 luciferase transcript signal against matched *Pfpcna* transcript signal intensity allowed the overall effect of 5′ sequence deletion on the steady-state level of luciferase mRNA accumulation to be compared to Δ1, with apparent reductions of 28%, 43%, and 96% for Δ2, Δ3, and ΔKO, respectively. For the 3′ flanking series, there appeared to be a distinct “all-or-nothing effect” (Fig. 2b). Steady-state luciferase transcript levels remained essentially unchanged from that of Δ1 in transfectants Δ4 and Δ5, both containing the mapped polyadenylation sites, while deletion of these sites in transfectants Δ6 and Δ7 yielded no observable luciferase transcript.

As expected from the Northern blot data, the maximal peak in luciferase activity was reported in the mature trophozoite/schizont (T3) stage for Δ1, Δ2, Δ3, Δ4, and Δ5 (Fig. 2c). Transfectants ΔKO, Δ6, and Δ7 had no luciferase activity above background levels in any of the time points assayed, emphasizing the key role for the mapped transcription start and polyadenylation sites that are missing in these constructs. While deletions in the 5′ series (Δ2 and Δ3) were accompanied by stepped reductions in the absolute level of luciferase activity in T3 (by 48% and 75%, respectively), no apparent difference was observed at the same time point in the 3′ series (Δ4 and Δ5) when compared to Δ1. One-way analysis of variants of the reduced luciferase activities in the 5′ deletion series revealed no significant differences in the T1 and T2 time points (*p* = 0.525 and *p* = 0.307, respectively), while a significant difference was observed at the T3 stage (*p* = 0.004), with the significant difference being found between Δ1 and Δ3 using Tukey's posttests. All tests for significance between Δ1 and the 3′ series (Δ4 and Δ5) at all time points were insignificant.

### Nuclear factors bind regions demonstrated to function in *Pfpcna* promoter activity

**Fig. 3:**
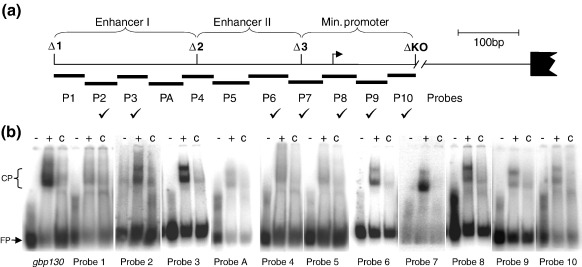
EMSA of distal 5′ *Pfpcna* flanking sequence. (a) A minimally overlapping tiling path of eleven 50–60mer double-stranded oligonucleotide probes covers the regions functionally analyzed in the promoter assay (see Table S1 for exact positions). The sites for the start of each clone (Δ1-ΔKO) are indicated with an annotation of the predicted function of the intermittent DNA sequences. Successful specific interaction with a nuclear extract is indicated by a ticked box below each probe. (b) EMSA showing free probe (−), probe with 5 μg of nuclear extract (+), or the same with a 200 molar excess of unlabeled homologous probe (C). The positions of the free probe (FP) and the complexed probe (CP) are indicated as is the probe used in each EMSA.

The *Pfpcna* promoter assays demonstrated the presence of two putative enhancer regions (− 1418 bp to − 1201 bp, and − 1200 bp to − 1031 bp) upstream of a newly refined minimal promoter region (− 1030 bp to − 840 bp). To explore the potential for nuclear trans-acting factor binding to the distal 5′ flanking regions identified, we generated a tiling path of 11 minimally overlapping 50–60mer double-stranded oligonucleotides (here termed probes) (Fig. 3a) and subjected it to electrophoretic mobility shift assays (EMSAs) with nuclear extracts from trophozoite-stage parasites. As control, *PfTBP*, a probe containing the TATA binding protein (TBP) binding site in the minimal promoter of *gbp130* (PF10_0159), was included in the analysis.^44^ To demonstrate the specificity of each interaction, we repeated the EMSAs with a 200-fold molar excess of homologous unlabeled probe to ensure complete ablation of any cis–trans interactions observed. Probes 2, 3, 6, 7, 8, 9, and 10 all showed evidence of nuclear factor(s) binding—a pattern of interactions suggesting that trans-acting factors bind to the most distal enhancer element and the entire minimal promoter region (Fig. 3b).

**Fig. S4:**
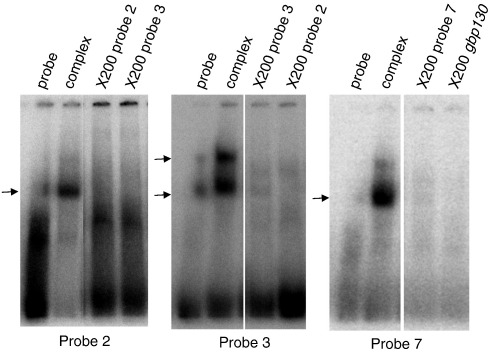
Cross competition EMSA. Probes 2, 3 and 7 were subjected to EMSA alone (probe) or with 5μg of nuclear extract (complex) and a 200–fold molar excess of the appropriate homologous or indicated heterologous unlabelled probe. The position of the respective probe-complex is indicated by an arrow.

**Fig. 4:**
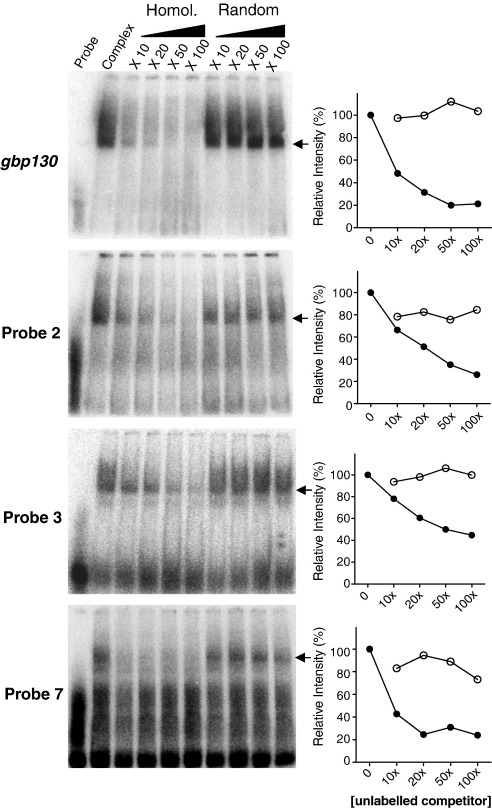
Specific and titratable cis–trans interactions in the distal 5′ regions of the *Pfpcna* promoter. The left-hand panels show EMSA with probes 2, 3, and 7 of *Pfpcna* and the *gbp130* positive control alone (probe), or with 5 μg of trophozoite-stage nuclear extract (complex) and the indicated increasing-fold molar excess of homologous or heterologous random unlabeled probe. The intensity of the indicated complex formed in each lane is plotted as a proportion of the signal in the absence of any homologous (black circles) or heterologous (open circles) competitors in the right-hand panels.

To demonstrate a specific and titratable effect typical of a true cis–trans interaction, we took forward the *gbp130* probe and probes 2, 3, and 7 for a more complete competition assay. The latter probes were chosen as (i) they gave strong, readily reproducible evidence of complex formation comparable to the control, and (ii) the complex formed was completely ablated upon competition with an excess of unlabeled homologous probe. Trophozoite nuclear extracts were preincubated with increasing molar excess of unlabeled homologous or nonhomologous (random sequence) probes before the addition of the labeled probe. The signal intensity of the bound complex was then determined as a proportion of the signal in the absence of any competition (Fig. 4). In all cases, titratable inhibition of complex formation was demonstrated using the homologous probe, with the specificity of this interaction shown by the absence of a comparable effect using increasing molar excess of the nonhomologous probe. Given that probes 2 and 3 are located within the same putative enhancer element and that probe 7 and *gbp130* probes are from comparable regions within their respective minimal promoters, cross-competition assays were carried out with these pairs of probes. The complexes formed by probes 2 and 3 were effectively competed by a 100 molar excess of both the homologous probe and the corresponding nonhomologous probe (Fig. S4), suggesting that the same trans-acting factor binds probes 2 and 3. Similarly, probe 7 complex formation was abolished by the addition of a 100 molar excess of the *gbp130* probe (Fig. S4).

**Fig. 5:**
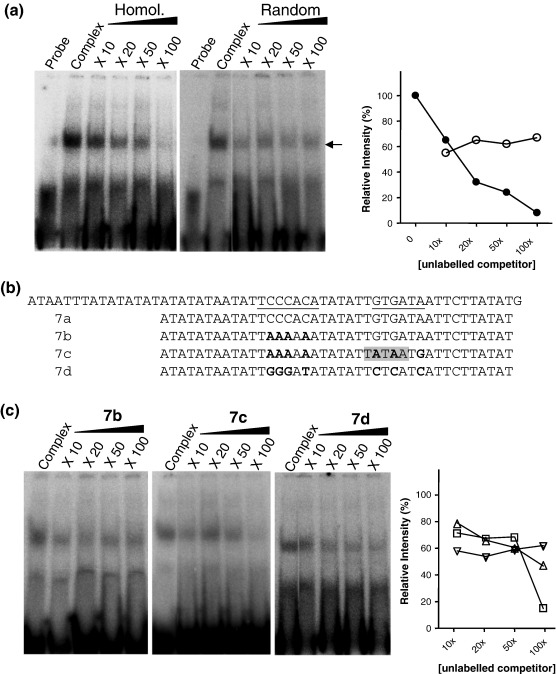
EMSA of modified probe 7. (a) The left-hand panels show EMSA with probe 7a alone (probe) or with 5 μg of trophozoite-stage nuclear extract (complex) and increasing-fold molar excess of homologous or heterologous random unlabeled probe. The intensity of the indicated complex formed in each lane is plotted as a proportion of the signal in the absence of any homologous (black circles) or heterologous (open circles) competitors on the right-hand graph. (b) Comparison of the sequence of probe 7 (top line) with the minimally refined probe 7a and versions thereof introducing site-specific mutations (in boldface) in the underlined sequences of interest. Sequence TCCCACA is the reverse complement of TGTGGGA, a variant of motif PM29.2,^45^ with the cryptic TATA box introduced in probe 7c highlighted in gray. (c) Competition EMSA for probe 7a complex formation in the presence of an increasing-fold molar excess of mutated probes 7b, 7c, and 7d. The intensity of the complex formed in each lane is plotted as a proportion of the signal in the absence of any competitor (probe alone). Probe 7b (inverted triangle), probe 7c (square), and probe 7d (triangle).

Probe 7 lies between 37 bp and 93 bp upstream of the mapped transcription start site, a position comparable to that demonstrated for *gbp130* and *kahrp* (PFB0100c), the site of preinitiation complex formation over the minimal promoter region.^44^ The design of probe 7 was refined to give a probe lying between 40 bp and 80 bp upstream of the transcription start site entirely within the mapped minimal promoter for *Pfpcna*. This new probe, designated probe 7a, was shown to bind the trophozoite nuclear protein(s), with the interaction being both specific and titratable (Fig. 5a). A series of mutations was introduced into this sequence to give probes 7b, 7c, and 7d (Fig. 5b). These mutations targeted two sequences shown by sequence comparison to share a level of homology to the cis-acting binding site for TBP and a novel motif (PM29.2) identified in *in silico* studies of overrepresented motifs in *P. falciparum* gene flanking sequences.^44,45^ Increasing molar excess of these unlabeled mutated probes was used to establish their potential to inhibit the interaction of probe 7a with trophozoite-stage nuclear protein(s) (Fig. 5c). While some reduction in the signal intensity of the bound complex formed in the presence of probe 7b was observed, this effect was clearly not titratable, with evidence of complex formation still evident on the addition of a 100-fold molar excess of unlabeled probe. A high molar excess of probe 7c, however, appears to considerably reduce probe 7a complex formation—an interesting observation as the mutations introduce a cryptic consensus *PfTBP* recognition site. Mutation of this cryptic site in probe 7d appears to restore probe 7a complex formation in the presence of a 100-fold molar excess of this unlabeled heterologous probe.

### Sublethal doses of histone deacetylase inhibitors do not release the temporal inhibition of *Pfpcna* expression in ring-stage parasites

Type I/II histone deacetylase (HDAC) inhibitors have been demonstrated to rapidly induce a profound dysregulation of the tight temporal control of transcription in the IDC, with evidence of histone hyperacetylation, morphological changes typical of programmed cell death (PCD), and delay in IDC progression with failure to reinvade.^46–50^ Here we explore the use of sublethal doses of two related HDAC inhibitors—suberoylanilide hydroxamic acid (SAHA) and suberohydroxamic acid (SBHA)—to determine their effect on the absolute and temporal expressions of luciferase in the Δ1 transfectant, in particular whether the temporal repression of luciferase expression in ring-stage parasites can be lifted.

Dose–response curves for SAHA and SBHA inhibition of parasite growth were determined using the 3D7 parasite strain, with IC_50_ values of 1.43 ± 1.03 μM and 8.06 ± 1.08 μM for SAHA and SBHA, respectively, comparable to previously published results.^46,47^ Preliminary time-course experiments using concentrations of drugs comparable to IC_2_ and IC_5–7_ demonstrated that both SAHA (0.05 μM and 0.5 μM) and SBHA (1.2 μM and 2.2 μM) induce time-dependent and concentration-dependent effects on parasite progression through the IDC, while not significantly decreasing parasitemia over 48 h of drug exposure (data not shown). Parasite cultures tightly synchronized to early rings (0–6 hpi) were exposed to both drugs at both concentrations, with samples taken at T1 (ring, 10–16 hpi), T2 (trophozoite, 22–28 hpi), T3 (mature trophozoite/schizont, 34–42 hpi), and T4 (schizont/early ring, 44–6 hpi) through, and onto the next, IDC for assessment of parasitemia, parasite staging, and luciferase activity.

**Fig. S5:**
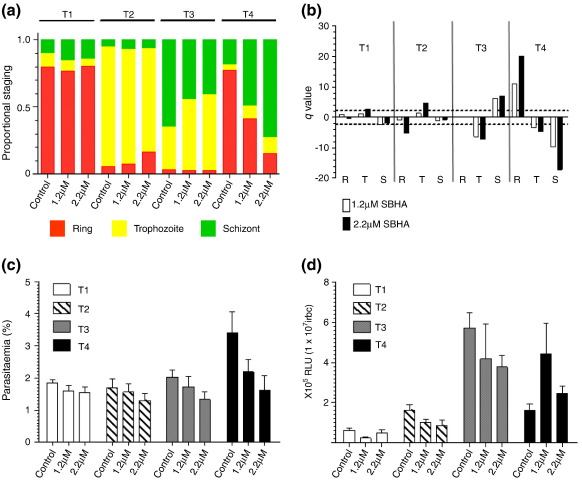
Treatment of Δ1 with sub-lethal doses of SBHA. (A) The mean proportion of each IDC morphological stage at each harvest timepoint (T1 rings, T2 early trophozoites, T3 mature trophozoites, T4 schizonts in untreated control) during the timecourse of treatment with either 1.2μM or 2.2μM SBHA or an untreated control. (B) Dunnetts test for proportions to determine significant differences (q value>2.21) in the proportion of each morphological stage (R, ring, T, trophozoite, S, schizont) in drug treated parasites (1.2μM or 2.2μM SAHA) compared to the untreated control. (C) Mean and SEM of parasitaemia of untreated control and SBHA treated cultures during timecouse of study. (D) Timecourse luciferase activity assay data for untreated control and SBHA-treated Δ1 expressed as relative light units x105 (RLU) per 1x107 infected red blood cells (irbc). Data represents mean and SEM of five independent paired measurements.

**Fig. 6:**
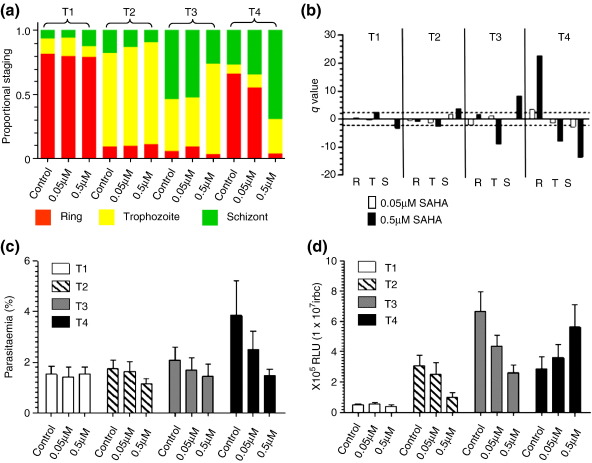
Treatment of Δ1 with sublethal doses of SAHA. (a) The mean proportion of each IDC morphological stage at each harvest time point (T1, ring; T2, early trophozoite; T3, mature trophozoite; T4, schizont in untreated control) during the time course of treatment with either 0.05 μM or 0.5 μM SAHA or the untreated control. (b) Dunett's test for proportions to determine significant differences (*q* > 2.21) for the proportion of each morphological stage (R, ring; T, trophozoite; S, schizont) in drug-treated parasites (0.05 μM or 0.5 μM SAHA) compared to the untreated control. *q* values of > + 2.21 and < − 2.21 (dotted lines) represent a significant decrease or increase in that morphological stage, respectively. (c) Mean and standard error of the mean of the parasitemia of untreated control and SAHA-treated cultures during the time course of the study. (d) Time-course luciferase activity assay data for untreated control and SAHA-treated Δ1 expressed as relative light units (RLU) × 10^5^ per 1 × 10^7^ infected red blood cells (irbc). Data represent the mean and standard error of the mean of five independent paired measurements.

For SAHA, assessment of parasite staging showed no significant difference in the relative proportion of each morphological stage during the first 24 h of drug treatment (T1 and T2) compared to the control without drug (Fig. 6a). From 24 h, however, we observed a significant difference in the relative proportion of the different morphological stages in T3 and T4 that increases with both time of exposure and drug concentration, apparently revealing a delay in progression of the IDC (Fig. 6b). This is exemplified by the significant reduction in the proportion of rings in T4 using 0.5 μM SAHA (IC_5_), with schizonts instead representing the predominant developmental stage present, supporting the evidence for no increase in parasitemia due to the failure to complete the IDC (Fig. 6c). Luciferase activity in the control cultures peaked, as expected, in T3 (mature trophozoite/schizont). Luciferase activity upon treatment with 0.5 μM SAHA is significantly reduced in T3 (*p* = 0.01), with lower levels also observed for treatment with 0.05 μM (IC_2_) SAHA and with levels of luciferase activity for both treatments higher than the control at T4 (Fig. 6d). The apparent shift in the peak of luciferase activity for SAHA-treated parasites correlates with the relative effect of the dose of the drug on parasite progression (i.e., the higher the dose of SAHA, the more pronounced is the delay in IDC, which then results in a greater shift in the peak of luciferase activity to the next time point in the harvest). Thus, while SAHA appears to alter the absolute pattern of temporal luciferase activity during T1–T4, the underlying effect of SAHA on the IDC means that the observed peak of luciferase activity in all samples is still maintained in the mature trophozoite/schizont stages. Importantly, for both concentrations of SAHA, there is no apparent induction of luciferase expression in ring-stage parasites. Treatment of parasite cultures with 1.2 μM and 2.2 μM SBHA, corresponding to IC_2_ and IC_7_, respectively, similarly demonstrates time-dependent and dose-dependent effects on IDC progression (Fig. S5). Again, the peak of luciferase activity is maintained in the mature trophozoite/schizont stage, with no evidence of induction of luciferase expression in ring-stage parasites.

We reasoned that the apparent lack of induction of luciferase expression in ring-stage parasites may result either from a delay in the effect of the sublethal doses of SAHA and SBHA within 24 h and/or from histone marks being “hard-wired” after being established in the previous cycle of the IDC. The latter supposition is supported by evidence indicating that commitment to sexual development is made in a prior cycle of the IDC and by evidence of epigenetic “tagging” of the active variant of the *var* multigene family being expressed in a subsequent IDC.^51,52^ To this end, we carried out a second time-course analysis where SAHA and SBHA were added to mature trophozoites/schizonts and harvests were taken at two time points (+ 24 h and + 48 h) in the subsequent IDC.

**Fig. S6:**
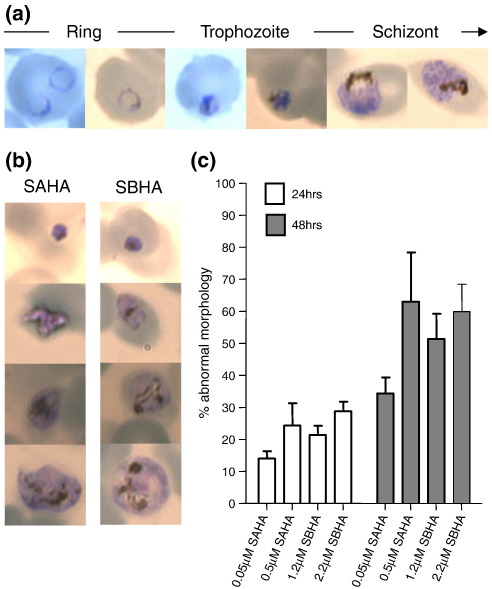
SAHA and SBHA treatment affects the normal morphological development of intraerythrocytic *P. falciparum*. (A) Giemsa-stained thin smears of untreated *P. falciparum* infected erythrocytes illustrating the normal progressive morphology during intraerythrocytic development. (B) Giemsa-stained thin smears of 0.5μM SAHA (left column) and 2.2μM SBHA treated *P. falciparum* infected erythrocytes illustrating the poor morphology of mature stage parasites. (C) Timecourse assessment of the proportion (mean±StDev) of mature stage *P. falciparum* intraerythrocytic stages showing poor morphology using the indicated drug treatment (n=3).

**Fig. 7:**
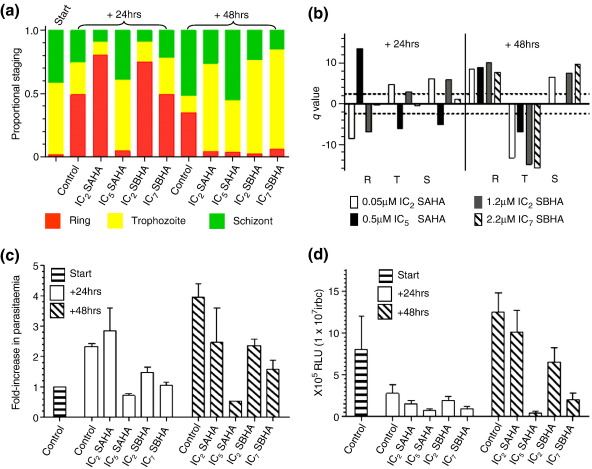
Treatment of Δ1 with sublethal doses of SAHA and SBHA in a previous cycle of IDC. (a) The mean proportion of each IDC morphological stage at each harvest time point (start, + 24 h, and + 48 h) during the time course of treatment with two different concentrations of SAHA and SBHA. (b) Dunett's test for proportions to determine significant differences (*q* > 2.21) in the proportion of each morphological stage (R, ring; T, trophozoite; S, schizont) in drug-treated parasites (see key) compared to the untreated control. See also the legend to Fig. 6b. (c) Mean and standard error of the mean of the fold increase in the parasitemia (compared to start) of untreated control and SAHA/SBHA-treated cultures during the time course of the study. (d) Time-course luciferase activity assay data for untreated control and SAHA/SBHA-treated Δ1 expressed as relative light units (RLU) × 10^5^ per 1 × 10^7^ infected red blood cells (irbc). Data represent the mean and standard error of the mean of five independent paired measurements.

The untreated control cultures showed the expected temporal pattern of morphological development with a 2-fold to 3-fold increase in parasitemia upon reinvasion (Fig. 7a–c). The luciferase activity peaked at the + 48 h time point in mature trophozoites/schizonts, with the increase in activity corresponding to the observed increase in parasitemia (Fig. 7c and d). The SAHA-treated and SBHA-treated cultures both show dose-dependent and time-dependent effects on the relative proportion of staging, replicating the previously described delay in IDC progression (Fig. 7a). Untreated cultures mature normally, with a small proportion undergoing a second round of reinvasion, as evidenced by the fold increase in parasitemia and the increased proportion of rings at + 48 h (Fig. 7c). Except for the higher dose of SAHA treatment, cultures subjected to drug treatments showed an increase in parasitemia over 24 h, although at a rate lower than that of the untreated control, but did not undergo a second round of invasion and remained predominantly in the mature stages of parasite development. The luciferase activities for the IC_2_-treated cultures were higher in the 48-h time point, although at a lower level than the control. This presumably reflects reduced parasite numbers and PCD, as evidenced by the poor morphology of these mature parasites at between 30% and 60% (SAHA, 35 ± 5%; SBHA, 51 ± 9%) (Fig. S6). The higher dose treatment of both drugs significantly decreased luciferase activities (*p* = 0.01) at + 48 h, again reflecting a significant reduction in parasitemia (*p* = 0.01) and an increase in the proportion of mature intraerythrocytic parasites with poor morphology to between 52% and 74% (SAHA, 63 ± 11%; SBHA, 65 ± 7%) . In all cases, however, irrespective of the drug or concentration applied, we were unable to induce luciferase expression in ring-stage parasites.

## Discussion

Here we have described perhaps the most complete study in *P. falciparum* that investigates the molecular mechanisms that control the absolute and temporal control of a prototypical housekeeping gene during intraerythrocytic development. Using 1418 bp and 647 bp of matching 5′ and 3′ flanking sequences from *Pfpcna*, we were able to reconstitute all aspects of the control of gene expression for an integrated luciferase reporter gene, emphasizing this approach in developing best practices in investigating gene expression in *P. falciparum*. Reconstituting the correct absolute and temporal patterns of mRNA and protein expression, as well as the 5′ and 3′ limits of the luciferase transcript in the Δ1 integrant, indicates that this parasite represents a model for the molecular control of expression for an integrated reporter cassette directly analogous to that of the endogenous gene. Using this system, we were able to refine the minimal promoter region and to additionally characterize two putative 5′ distal enhancer elements, as well as to describe specific and titratable nuclear trans-acting factor interactions with these elements. Analysis of the sequences of the probes used in the EMSA study reveals three interesting findings. First, probe 7, which lies within the minimal promoter region, potentially contains a cis-acting element for the TBP of the TFIID complex.^44,53,54^ Specific annotation of the TATAA motif in *P. falciparum* is difficult given the extreme AT bias of intergenic sequences, often in excess of 90%.^2^ However, functional evidence for binding was suggested initially by the TBP binding site of *gbp130* effectively competing with probe 7 complex formation and was then strengthened further when introduction of a cryptic TBP site in probe 7c led to effective competition of probe 7a complex formation—an effect that was ablated when the cryptic site was mutated in probe 7d. Second, probe 7 also contains a putative PM29.2 motif (TGTGTGA) in the form of TGTGGGA, a motif found to be significantly overrepresented in the 5′ flanking sequences of genes sharing the same temporal pattern of *Pfpcna* expression and/or Gene Ontology annotation in the replisome.^45,55,56^ Mutation of this motif in probes 7b and 7d could not effectively compete with probe 7a formation at a 100 molar excess, although a specific titratable effect was difficult to achieve, as probe 7a presumably interacts with more than one trans-acting factor. The third finding was the identification of a novel putative cis-acting sequence, TGAA(AT)(AT)GG, present as a single copy in each of probes 2 and 3, although inverted with respect to one another. While novel, the evidence that probes 2 and 3 can effectively compete with the formation of the other's complex and that both probes lie within the same putative enhancer region responsible for some 50% of promoter activity do suggest some functional role.

Mapping of the 3′ polyadenylation sites for *Pfpcna* and luciferase genes identified three sites that share consensus sequences with other eukaryotes—a distinctly different picture when compared to 5′ regions where *Plasmodium* spp. appear to lack any homology to cis-acting sequences present in other eukaryotes.^16^ The three AATAA motifs, each closely followed by a U/GU-rich region within 20–40 bp, are identical with the only other polyadenylation sites functionally characterized in *P. falciparum*, those of *pfs25*.^43^ The functions of these sites in *pfs25*, however, were only established in a heterologous transfection system using the chicken malarial parasite *P. gallinaceum*.^43^ Here we have shown that these consensus polyadenylation sequences operate, as expected, in a homologous transfection system, and we have demonstrated further their fundamental “all-or-nothing” role in maintaining transcript stability.

Perhaps the most important finding of this study, however, was that while our genetic deletion and HDAC inhibitor studies show that we can affect the absolute level of *Pfpcna* expression, no effect on the temporal control of *Pfpcna* expression was achieved. While the temporal control of a transfected reporter cassette does rely on appropriate nucleosome assembly,^27,28^ a detailed analysis of the role of histone modifications in directing this temporal control still remains to be established.^17,57^ Here we addressed whether sublethal doses of the HDAC inhibitors SAHA and SBHA could induce a transcriptional permissive state in any other part of IDC development, and specifically whether the temporal repression of *Pfpcna* expression in ring stages could be released. While able to demonstrate effects on IDC progression, induction of morphology typical of PCD, and failure for reinvasion, which are all established effects of HDAC treatment, we found no evidence for an altered temporal profile of *Pfpcna* expression—this in spite of using two different drugs at two different concentrations and inducting treatment at two different points during the IDC.

Together, these findings suggest that temporal control of *Pfpcna* expression during the IDC is not solely mediated by either genetic elements adjacent to the promoter or epigenetic factors—both of which, however, are important mechanisms for the control of the absolute level of expression. So how then is temporal expression of *Pfpcna* controlled? Recent data from three published studies, taken together with the data shown here, suggest that posttranscriptional mechanisms may drive the temporal expression of *Pfpcna*. These published studies indicate the following: (i) a global program of RNA polymerase II activation during the IDC, with the peak of activity in mature trophozoites coinciding with the replication of the genomic template;^58^ (ii) a similarly global program of the increasing stability of mRNA transcripts during IDC progression;^15^ and (iii) key components of the preinitiation complex poised on promoters throughout the IDC.^59^ Thus, in ring-stage parasites, where *Pfpcna* has been demonstrated to have a low level of promoter activity and the mRNA transcript has been demonstrated to have a short half-life (2.55 min),^6,15,36^ these mechanisms together would account for the low steady-state levels of transcript and the low level of protein expressed. In mature trophozoites, *Pfpcna* promoter activity has been shown to dramatically increase in line with the known global activation of RNA polymerase II at this stage of the IDC, which, together with the dramatic increase in mRNA stability (half-life, between 53.4 min and 78.3 min), would account for the high steady-state mRNA levels and the corresponding increased levels in protein expression.^6,15,36^

**Fig. S7:**
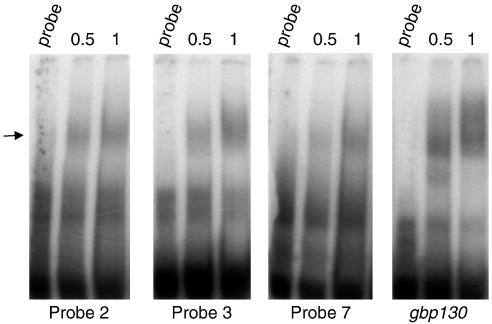
EMSA using ring stage nuclear extracts. Probes 2, 3, 7 and *gbp*130 were subjected to EMSA alone (probe) or with 0.5 or 1μg of ring-stage nuclear extract. The position of the respective probe-complex is indicated by the arrow.

We suggest that deletion of *Pfpcna* 5′ flanking distal elements prevents the binding of trans-acting factors that recruit and/or stabilize preinitiation complex formation. EMSA using ring and trophozoite nuclear extracts indicates that probes 2, 3, and 7 bind a nuclear trans-acting factor (Fig. S7), data that support an apparent pre-initiation complex (PIC) formation throughout the IDC. The “all-or-nothing” effect of the deletion of the polyadenylation sites in pΔ6 and pΔ7 is presumably accounted for by the role that these sequences play in mediating mRNA stability. Thus, while the genetic and epigenetic factors investigated here could readily account for altered absolute levels of activity, none would overcome the global mechanisms operating at the level of RNA polymerase II activity and mRNA stability. While apparently a novel means to drive the temporal cascade of gene expression during the IDC, recent evidence has suggested that *P. falciparum* RNA polymerase II is an intrinsically novel protein,^60^ exhibiting previously unreported intraspecies plasticity in the number and composition of its carboxy-terminal repeats governing the plethora of RNA-polymerase-II–mediated activities. These finding support a fundamental role for posttranscriptional mechanisms in governing the temporal expression of potentially hundreds of genes during a stage of parasite development responsible for the etiology of this devastating disease.

## Materials and Methods

### Transfection constructs

All luciferase reporter cassettes were replaced as ApaI-PstI fragments into a similarly restricted pDC1600crtGFPAttP plasmid (a kind gift of D. Fidock, Columbia University, New York, NY), replacing the existing green fluorescent protein reporter construct.^33^ The original *Pfpcna* luciferase reporter cassette pPLH1^21^ was modified to generate an intermediate plasmid, pmPLP1, where the luciferase gene was flanked by 1418 bp of 5′ flanking sequence and 647 bp of 3′ flanking sequence from *Pfpcna* (PF13_0328). Here the *Pfpcna* 5′ flanking sequence is modified by the addition of a unique 8-bp sequence tag, consisting of the NotI restriction site 840 bp upstream of the luciferase gene, to differentiate this sequence from that flanking the endogenous gene. An ApaI-PstI fragment from pmPLP1 was subcloned into the pDCAttP host plasmid to generate plasmid Δ1.

The 5′ deletion series was prepared by PCR upstream of the NotI sequence tag to incorporate 1200 bp (Δ2), 1030 bp (Δ3), and 860 bp (ΔKO) of 5′ flanking sequence (flanked with unique ApaI and NotI restriction sites to facilitate ligation) into iterations of the pmPLP1 plasmid and then subcloned into the pDCAttP plasmid. The 3′ deletion plasmids were generated by PCR over a unique EcoRV site at the 3′ end of the luciferase gene in pmPLP1 to generate products containing 130 bp (Δ4), 350 bp (Δ5), 494 bp (Δ6), and 591 bp (Δ7) of the *Pfpcna* 3′ flanking sequence. These were directly cloned back into EcoRV-PstI-restricted Δ1. A detailed subcloning protocol is available in Fig. S2.

### *P. falciparum* culture and transfection

The AHE1 clone (Dd2^*att*^^B^) of *P. falciparum* was provided by D. Fidock.^33^ This is a clone of Dd2 where the *att*B site for mycobacteriophage *bxb1* integration into the genome of *M. smegmatis* has been inserted into the *cg*6 locus of chromosome 7. The presence of this site is maintained by 5 nM WR99210 drug selection. Parasite cultures were maintained as previously described using successive treatment with 5% sorbitol^61^ to maintain synchronization. Staging and parasitemia of parasite cultures were confirmed by light microscopy of Giemsa-stained thin blood smears. Parasitemia was determined by the proportion of infected erythrocytes from a count of 1000–2000 erythrocytes, with staging determined from a count of at least 200 infected erythrocytes. Transfections of *P. falciparum* and subsequent drug selection were carried out as described by Nkrumah *et al.* using 40 μg each of the appropriate luciferase reporter plasmid and the pINT plasmid (encoding the *bxb1* integrase protein). At least two independent clones for each plasmid were prepared. Isolation and size fractionation of genomic DNA and RNA were carried out as previously described.^62,63^

### 5′ and 3′ RACE

Parasite RNA (1 μg) from trophozoite-stage parasites was used for first-strand cDNA synthesis for both 5′ and 3′ RACE protocols. The 5′ RACE-RLM™ System kit (Invitrogen) was used, according to the manufacturer's instructions, with first-strand cDNA synthesis from the GSP1 primer (5′-TCAAAAGTTATTTTTAAAGATATAAAT GATAATGTGC-3′) located 542 bp upstream of the luciferase open reading frame. Nested PCR on the first-strand cDNA was then performed using the first GSP2 (5′-TTTATAAATGTTTCTTAATGTATAACTTATGTG-3′) and then GSP3 (5′-AAATCAAAATGATAAAGCGGCCGC-3′) with the supplied 5′ RACE system AUAP primer. The use of GSP3 targeted to the NotI sequence tag ensured amplification only of the 5′ end of the luciferase mRNA. 3′ RACE was performed, using the GeneRacer™ kit (Invitrogen) according to the manufacturer's instructions, with first-strand cDNA synthesis from an oligo(dT) primer. Nested PCR on the first-strand cDNA was carried out for both luciferase and *Pfpcna* transcripts using pairs of primers designed at the 3′ ends of each open reading frame and the supplied 3′ GeneRacer™ primer (Invitrogen) and GeneRacer™ nested primer (Invitrogen): Luc_out_ (5′-CGAAGTACCGAAAGGTCTTACCGG-3′) and Luc_in_ (5′-AGGCCAAGAAGGGCGGAAAGTCC-3′), *Pfpcna*_out_ (5′-AACATTTTAGCAGATGTCGTTGTCTTAGG-3′) and *Pfpcna*_in_ (5′-GATACCTCCCCTGATTCAGATTCAGATACC-3′), where the second primer (X_in_) represents the oligonucleotide used in the nested PCR. 5′ and 3′ RACE products were subcloned into the pCRII-Blunt-TOPO® vector (Invitrogen) and sent for commercial sequencing (Eurofins Mwg). Sequence comparisons were carried out using ClustalN†.

### Luciferase assays

A standard luciferase assay protocol based on previous protocols^21^ was followed using a commercially available Luciferase Assay kit (Promega). For each assay, two 100-μl samples of packed infected erythrocytes were removed from synchronized *P. falciparum* culture at approximately 1% parasitemia. Parasites were isolated by lysis in 1× phosphate-buffered saline/0.1% saponin and subjected to freeze–thaw lysis in the supplied reporter lysis buffer. Five microliters of the cleared supernatant was added to 50 μl of the supplied luciferase substrate reagent assay, and luciferase activity was measured using the LumiCount™ (Packard) apparatus controlled using PlateReader™ version 3 (Packard). All data are reported as arbitrary light units per 1 × 10^7^ parasites, and the mean of at least five pairs of independent experiments was analyzed using one-way analysis of variants on MiniTab version 14.

### IC_50_ determination

IC_50_ determination was carried out as described by Desjardins *et al.*,^64^ as modified by Bennett *et al.*^65^ Stocks (10 mM) of SAHA and SBHA (kind gifts of K. Andrews, Queensland Institute of Medical Research) were prepared in dimethyl sulfoxide, and a serial 3-fold dilution was carried out on a 96-well plate starting from an initial well concentration of 100 μM. *P. falciparum* culture was added to each well to give a 2% final initial parasitemia at 2% hematocrit. The plates were incubated for 48 h in a humidified incubator flushed every 24 h with 1% oxygen, 3% CO_2_, and 96% nitrogen. Growth inhibition was assessed by SYBR I green incorporation and plotted as a percentage of growth against untreated parasites (100%), and *P. falciparum* culture was exposed to a supralethal dose of artemether (0%). Data from two independent plates containing triplicate wells for each treatment were analyzed using GraphPad Prism (version 5) software to determine the IC_50_ concentration from the sigmoidal dose–response curve.

### Electrophoretic mobility shift assays

Nuclear proteins were isolated using a modified procedure of the protocol published by Lanzer *et al.*^66^ First, cytosolic proteins were extracted using the NE-PER® (Pierce) nuclear and cytoplasmic extraction reagents according to the manufacturer's instructions. Protein extraction of the nuclei pellet was performed using low-salt buffer to remove excess cytosolic proteins, followed by high-salt buffer upon incubation on ice, releasing nuclear proteins from the nuclei. The integrity of the nuclear protein extracts was confirmed by polyacrylamide gel electrophoresis and Coomassie blue staining. Protein content was measured using the NanoDrop1000 (Thermo Scientific). Five micrograms of nuclear extract was incubated in 20 μl of 1× EMSA binding buffer [20 mM Hepes (pH 7.9), 10 mM NaCl, 5 mM MgCl, 1 mM dithiothreitol, 0.25 mg/ml bovine serum albumin, and 5% glycerol] containing 500 ng of poly(dAdT) as nonspecific double-stranded competitor DNA, 50 fmol of single-stranded oligonucleotide probe (N_18_), and 1× Halt™ Protease Inhibitor Cocktail (Pierce) for 10 min at room temperature prior to addition of 2.5 fmol of γ-[^32^P] end-radiolabeled double-stranded oligonucleotide probes and incubation for 15 min at room temperature. Reactions were size-fractionated on 6% polyacrylamide gels in 0.5% Tris/borate/EDTA and exposed to a phosphor screen for up to 2 days. The image was captured using the Cyclone storage phosphor screen apparatus (Packard) and analyzed using the OptiQuant software (Packard). For competition experiments, the nuclear extract was incubated with varying molar excess of unlabeled double-stranded oligonucleotide probe (homologous or heterologous) for 10 min in binding buffer before the addition of the labeled probe and further incubation for 15 min at room temperature. A probe designed against the known *gbp130* (PF10_0159) *PfTBP* binding site (5′-TTATATTATGAATAAATGTAA GCAGAAAAGGAATGGTGT) was used as a positive control.^44^ Signal intensity analysis was performed on Photoshop CS3 following discarding of color data to leave a gray-scale image. The mean intensity value of a defined region was captured, normalized against the background, and plotted using Prism GraphPad (version 5).

The following are the supplementary materials related to this article.

Supplementary Table

## Footnotes

† http://srs.ebi.ac.uk
